# Intercepting Dementia: Awareness and Innovation as Key Tools

**DOI:** 10.3389/fnagi.2021.730727

**Published:** 2021-10-13

**Authors:** Emanuele Brai, Alessandro Tonacci, Victòria Brugada-Ramentol, Federica D’Andrea, Lavinia Alberi

**Affiliations:** ^1^Brain Fit4Life, Fribourg, Switzerland; ^2^Laboratory of Neuroplasticity, Department of Pharmaceutical Sciences, University of Piemonte Orientale, Novara, Italy; ^3^Institute of Clinical Physiology, National Research Council of Italy (IFC-CNR), Pisa, Italy; ^4^Virtuleap, Lisbon, Portugal; ^5^School of Biomedical Sciences, University of West London, London, United Kingdom; ^6^Swiss Integrative Centre for Human Health (SICHH), Fribourg, Switzerland; ^7^Department of Oncology, Microbiology and Immunology, Faculty of Science and Medicine, University of Fribourg, Fribourg, Switzerland

**Keywords:** dementia, stigma & awareness, innovation, technologies, prevention, advocacy

## Abstract

Dementia is a common feature of several age-related brain diseases, leading to a progressive cognitive decline. Due to a growing aging rate, dementia-related disorders currently affect around 50 million people worldwide and by 2050 this number is expected to reach 150 million. Additionally to patients, these neurodegenerative pathologies have a strong impact on family members, caretakers, and other health professionals, therefore representing a public health burden that in 2020 accounted for over 1 trillion USD and is projected to nearly double in the next decade. To overcome this devastating condition, many organizations and collaborative networks sustain that only a complete understanding of dementia in its different characteristics can drive the scientific community towards the development of effective therapeutic approaches aiming at preventing its onset and halting its progression.In this work, we discuss two topics that represent fundamental resources in fighting dementia: (i) the importance of raising awareness about this condition to avoid stigma and gauging investment; and (ii) the introduction of novel screening measures to prevent and potentially revert cognitive decline. Finally, we discern how knowledge-based advocacy will help the rollout of clinical trials and the development of novel and timely pharmacological interventions.

## Introduction

In 2020, the last report from the United Nations revealed that globally there were 727 million people aged 65 or more. This number of older adults is progressively rising and is predicted to reach 1.5 billion by 2050 (United Nations, Department of Economic and Social Affairs and Population Division, 2020). The demographic escalation of the senior population leads to a dichotomy in our society, indicating a longer life expectancy due to remarkable advances in the socio-economic and healthcare system, but also an increment of age-related disorders, such as dementias (Brown, 2015; Franceschi et al., 2018; Kehler, 2019). The term “dementia” encompasses several neurodegenerative disorders, of which Alzheimer’s disease (AD) is the most common form. AD remains largely untreatable^1^, with the exception of the recently approved drug Aduhelm for subjects with mild cognitive impairment (MCI)^2^ a prodromal state to dementia. At present, there are 50 million patients living with dementia worldwide and, by 2050, this number is expected to exceed 150 million without the introduction of effective therapies (United Nations, Department of Economic and Social Affairs and Population Division, 2020). To address such a large segment of society, the direct and indirect costs will inevitably increase with the estimate of reaching 2 trillion USD by 2030 (El-Hayek et al., 2019; Siva, 2021). Other serious concerns, often overlooked, are misinformation and stigma about dementia that still represent major barriers in seeking medical consultation, examinations, and treatment. Consequently, these aspects can potentially delay diagnosis, exacerbate the psychophysical symptoms, mislead proper investment in research and clinical settings, as well as shortages and poor quality of health services to assist people with dementia and their carers. To counteract this alarming scenario, multiple actions are put in place, spanning from increased research activities to the dissemination of advocacy events, to acknowledge dementia as a global health crisis. Thus, underlying the need for international collaborations and for an improved societal understanding of this condition. For instance, this situation is becoming a high priority matter in the agenda of many national agencies, including the Swiss Alzheimer’s Association^3^ or, at the international scale, Alzheimer’s Disease International (ADI)^4^, Alzheimer’s Association US^5^, and growing activists groups such as BrainFit4Life^6^ and Brain Health Scotland^7^, with a strong social media footprint. All these organizations seek to represent a knowledge reference based on the topic of brain aging by providing appropriate information on dementia for patients, carers, healthcare professionals but also, more generally, the lay public. Recently, ADI sponsored the campaign “Let’s Talk about Dementia” to promote an open dialogue and disseminate public awareness on this syndrome to challenge common prejudice and stigmatization of patients and their families (ADI-World Alzheimer’s Month, 2019), while this year’s initiative “Warning Signs of Dementia” highlights the importance of a timely diagnosis to detect dementia in its early phase.

Moreover, during the last World Economic Forum, a new initiative called Davos Alzheimer’s Collaborative (DAC) highlighted the urgency of speeding up the process of discovery, screening, and development of efficient therapeutic strategies for curing AD (Siva, 2021). To successfully achieve these goals, a cross-disciplinary intervention is required and needs to involve and connect different bodies, including policymakers, academia, private and public research sectors, hospitals, caregivers associations, and advocacy groups (El-Hayek et al., 2019; Steiner et al., 2020; Siva, 2021). In line with the necessity of a more comprehensive approach, preventive methodologies are undergoing a fervent transformation, as demonstrated by an increasing focus on identifying innovative predictive tools. Novel digital technologies, such as wearable devices (smartwatches, fitness trackers) and mobile apps, have the ability to collect real-time physiological parameters, like sleep quality, blood oxygenation, and cardiac rate, as well as cognitive performance relevant for detecting preclinical dementia (Westerberg et al., 2010; Mancini et al., 2016; Piau et al., 2019; Gielis et al., 2021). Additionally, virtual reality (VR) and home-based systems are gradually attracting interest in the neuroscience field since their application allows the detection of minor cognitive changes and locomotor disabilities that remain otherwise unnoticed with the current methods (Zygouris et al., 2017; Piau et al., 2019).

The introduction of next generation technologies in studying and monitoring neurodegenerative deficits offers the unparalleled advantage of acquiring a large amount of longitudinal data in a fast-paced manner and providing a daily follow-up to the end-users. Moreover, it provides valuable information for specialists which can simultaneously analyze multiple biometrics and rapidly intervene, if necessary. Therefore, these records can be employed to further characterize peculiar signs occurring in the progressive phases of dementia and then help researchers to understand the pathology development, accelerate biomarkers identification, and continuously adapt technologies to the patient’s needs. Consistent with these observations, many of the metrics tracked with digital technologies are influenced by lifestyle (Kivipelto et al., 2018; Rosenberg et al., 2020). A recent study from Livingston and colleagues supports the link between lifestyle and dementia, indicating 12 risk factors as potential contributors for this condition (Livingston et al., 2020). In particular, the authors suggest that the modification of some habits, such as education, smoking, diet, physical activity, etc., may have a strong impact in preventing 40% of dementia cases worldwide (Livingston et al., 2020).

Here, we provide insights on relevant topics that, now more than ever, are at the forefront in the challenging path towards a world without dementia. In particular, we discuss on: (i) stigma around dementia; and (ii) the use of novel technologies to detect early signs of dementia and potentially prevent or reduce and delay major symptoms. Finally, we propose that improving awareness around dementia and adopting digital innovations may boost the quality and completeness of research studies and clinical trials, and finally intercept dementia before its progression becomes irreversible.

## Increasing Awareness to Defeat Stigma Around Dementia

In dementia-related disorders, as in other chronic debilitating pathologies, the diminished self-sufficiency of the patients is counterbalanced by the progressive dependency from others, such as family members, caregivers, and health practitioners. The degree of assistance may depend upon the stage of the disease, the presence of comorbidities, and the availability of family members that can help their relatives. This gradual psychophysical disability, primarily experienced by people with dementia, can also alter the wellbeing of the caring categories, which are exposed to long-lasting stressful situations difficult to handle and possibly detrimental for appropriate management of the illness, but also of their quality of life (Gove et al., 2016; Fletcher, 2019; Kane et al., 2020). Although millions of people face every day, directly or indirectly, this devastating burden, the awareness, and understanding about dementia are still insufficient (Lion et al., 2020). Therefore, it is of vital importance to react to the initial shock due to the diagnosis and adopt effective measures to preserve the quality of life of patients and families for as long as possible. To achieve this, the society and governments play a key role by sustaining and guiding the involved individuals throughout the pathological process and providing specific care services with trained specialists including caregivers, general practitioners, neurologists, psychologists, geriatricians, as well as volunteers (Gove et al., 2016; Rewerska-Juśko and Rejdak, 2020). At the societal level, stigma and lack of knowledge around dementia impact on the socio-economic dynamics, influencing funding allocated to care and research domains as well as compromising clinical studies’ quality and completeness. To overcome this concern, many global initiatives are underlining the necessity of a constructive dialogue between various stakeholders, since only a broad collaboration can promote a full comprehension of dementia in its diverse facets (Gove et al., 2016; Hand, 2019; Kane et al., 2020), following the model of cancer research and care initiatives.

In line with this, the World Health Organization (WHO) addressed stigma towards people with dementia as a key policy priority and features in the World Health Organisation Global Action Plan^8^. In addition, the World Alzheimer Report 2019, published by ADI, describes the results obtained by the biggest survey on attitudes to dementia that involved approximately 70,000 participants from 155 countries and belonging to different categories, such as people affected by dementia, healthcare personnel, and lay public. Some of the collected answers highlighted a worrying picture, namely one in four people believe that dementia is an inevitable process. Moreover, 62% of professionals consider dementia as a normal component of aging, 48% of participants think that the cognitive function cannot ameliorate through medical intervention, and 78.2% of the interviewed are convinced to develop dementia^9^, thus perpetuating the pervasive myth that dementia is an intrinsic part of the aging process. This scenario can delay an appropriate medical evaluation and consequently preclude a prompt and precise diagnosis and care (Werner and Giveon, 2008), therefore explaining the recurrent predominant assumption that “nothing can be done” to avoid cognitive decline upon aging. Moreover, increasing evidence suggests that inaccurate information about the causes of dementia and the personal effects further instigate the fear of developing neurodegenerative diseases (Ebert et al., 2020).

Upon dementia diagnosis, the initial sense of fear and confusion can trigger, in patients and their families, a domino effect that undermines self-esteem, and could generate anxiety about external judgments, hence triggering isolation from others and social activities (Werner and Davidson, 2004; Werner, 2005; Riley et al., 2014; Fletcher, 2019; Kane et al., 2020; Low and Purwaningrum, 2020; Rewerska-Juśko and Rejdak, 2020). These negative feelings and behaviors can permanently destabilize plans for the future and also fuel the disease progression, exacerbating its symptoms and even lead to a premature loss of autonomy and also institutionalization.

Well-organized multidisciplinary actions are fundamental to promote a proper understanding of brain aging and dementia, thus eliciting a broader and accurate perception of this condition that may also contribute to the advancement of more appropriate preventive strategies, timely diagnosis, and effective treatments (Gove et al., 2016; Steiner et al., 2020). This shift would also modify the community mindset when interacting with people affected by dementia and their families, enabling a respectful and sensitive confrontation that would have a broadly beneficial effect, therefore positively ameliorating their feelings and wellbeing. Reducing frustration and isolation would also increase hope on others, the society and possibly improve some symptoms and trust in clinical research. Moreover, the general population can learn how avoiding discrimination towards dementia can have a positive reflection on the quality of life (Gove et al., 2016; Rewerska-Juśko and Rejdak, 2020).

These actions should involve governments, research centers, hospitals, care homes, and associations and target all segments of the population. Only cooperation among different organisms can stop the vicious cycle of stereotypes and revert public misconceptions around dementia and defeat discriminative behaviors on elderly patients and their family members, since inequity and intersectionality, also neglected in this context, are further exacerbated when linked to aging and related disorders (Bartlett et al., 2018; Holman and Walker, 2021). The positive outcome in reducing stigma would also benefit the integrity and accuracy of clinical trials since knowledge can stimulate a broader enrollment (including healthy subjects and patients) in research studies. In this case, favoring inclusion and diversity and consequently challenging unequal aging and intersectionality may play a fundamental role in drug development pipelines, since research projects can be better designed and performed, therefore increasing the success rate in finding efficient predictive and therapeutic strategies against dementia-related disorders.

## New Technologies for Early Detection and Prevention of Dementia

Currently, pathologies such as dementia lack effective pharmacological treatments to slow down or arrest cognitive decline progression. In some disorders, the presence of altered core biomarkers can be identified before cognitive impairment occurs, without representing a diagnostic tool. Therefore, determining reliable biomarkers (biological and digital) to detect cognitive decline during preclinical stages will broaden awareness and ultimately promote prevention.

In this direction, the continuous evolution and development of new technologies in many sectors of our society also occur in the brain research domain. Tech companies have been showing a growing interest toward the generation of “smart” devices for physiological monitoring and assessment of cognitive functions, both heavily affected in dementia-related disorders. The emerging technologies that hold great promise in the prognosis of neurodegenerative disorders cover a wide spectrum of applications, ranging from the development of wearable devices, such as fitness trackers, smartwatches, and mobile apps, to artificial intelligence platforms, VR, and game-based systems, purposely designed for early diagnosing, monitoring and treating dementia symptoms (Zygouris et al., 2017; Mandryk and Birk, 2019; Piau et al., 2019; Bloniecki et al., 2021; Gielis et al., 2021; Majnarić et al., 2021). Overall, digital tools provide a constant and extensive amount of data reflecting an individual’s health status. For instance, it is possible to collect information about heart and respiration rate, sleep quality, physical performance, or cognitive functions, which progressively deteriorate with aging and can warn of preclinical states of dementia (Westerberg et al., 2010; Chester and Rudolph, 2011; Mancini et al., 2016; Lucke et al., 2019).

VR systems are rapidly gaining attention for their application in assessing cognitive training tasks, since, compared to screen-based tools, portable VR devices provide a simultaneous and more complete array on different sensory stimulations, including visual and auditory inputs or locomotor activity (Sánchez-Vives and Slater, 2005), therefore offering a real-time readout on multiple cerebral functions (Zygouris et al., 2017). In addition, their acceptance can be higher due to low obtrusiveness, engaging design, and gamified configuration (Kaufman et al., 2016; Zygouris et al., 2017; Mandryk and Birk, 2019), as some evidence points towards the benefits of gamified scenarios in improving specific cognitive aspects (Anguera et al., 2013). VR environments provide ecologically valid scenarios, which at the same time offer the possibility to control experimental variables (Bohil et al., 2011). Furthermore, VR has already proven useful in the field of neurorehabilitation (Pérez-Marcos et al., 2018). Altogether, these observations suggest that cognitive testing through VR could give a complete and naturalistic setting to test for long-term cognitive changes. For instance, it has been proposed that digital biomarkers extracted from gameplay could reflect the user’s cognitive status (Gielis et al., 2021).

Beyond that, several user interfaces have been recently implemented to administer basic cognitive tests-such as the Montreal Cognitive Assessment (MoCA; Nasreddine et al., 2005), one of the most common screening methods used for the detection of MCI and AD, and normally conducted using “pen and paper”—in a computerized version that showed promising results to date (Berg et al., 2018). Another example indicating the expansion and contribution of new appliances in monitoring brain health is given by Bloniecki and coworkers, who developed a novel digital test to assess cognitive performance and validated its accuracy against the MoCA test (Bloniecki et al., 2021). These short examinations are reliable in distinguishing individuals with advanced cognitive impairment, but unable to detect earlier stages of deficit and insufficient to quantify subtle subjective cognitive complaints. Using computerized or VR mental evaluation tools would produce a more sensitive interpretation, as it would not only provide cognitive but also behavioral parameters (e.g., reaction times, attention, dexterity, and decision-making; Valladares-Rodriguez et al., 2019). However, further studies are needed to understand the power of these measures as early predictors of intellectual dysfunction.

Another advantage offered by the technological innovation applied to the neuroscience field is the possibility to reduce or eliminate the intra- and inter-operator “bias” in test administration, to support the delivery of self-based monitoring tools in the framework of “p4 medicine”, and to enhance the predictive power of the “classical methods” used to detect dementia-related pathologies. Overall, the application of novel technologies offers healthcare professionals a more accurate setting for their diagnosis.

One of the main limitations linked to the regular utilization of digital devices is represented by the age of the end-users. Even though the number of subjects familiar with modern technologies is rapidly increasing, the knowledge about the availability and the use of these applications is still poor among older adults. Recently, further boosted by the COVID-19 pandemic, the research towards new, engaging easy-to-use instruments, could stimulate also in the neurology field the end-user curiosity and ultimately increase the acceptability among the elderly. Another limit is represented by the current absence of prospective studies addressing and evaluating the accuracy and specificity of novel technologies as reliable dementia predictors applied on a heterogeneous cohort of subjects (healthy controls and people affected by dementia with progressive severity) over a medium-long term period. This aspect requires a high priority realization, since, in this way, the initial promising outcomes obtained using digital biomarkers could promote their combination with other procedures, in a multimodal analysis approach, to better predict, monitor, and possibly prevent dementia (Turner et al., 2020; Young et al., 2020).

## Towards Better Clinical Trials Design and Development

**Figure 1:**
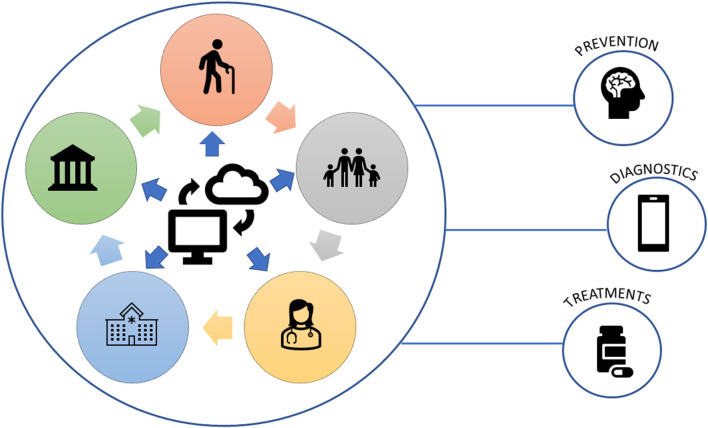
Networking and collaboration among different stakeholders: a fundamental chain in the improvement and identification of new preventive strategies, diagnostic tools, and effective treatments against dementia.

At present, there are no effective treatments to prevent and cure dementia-related disorders. However, in 2020 Cummings and colleagues reported that for Alzheimer’s disease there were more research studies compared to the previous year, therefore increasing the chance of identifying efficient therapeutic compounds. In particular, 121 drugs were under investigation at different trial phases and clustered into three categories based on their mechanism of action, i.e., (i) improve cognitive function; (ii) reduce neuropsychiatric and behavioral symptoms; and (iii) modify disease pathobiology (Cummings et al., 2020). Notably, in the latter group, one agent, Aducanumab, has been recently approved by the FDA, becoming the first disease-modifying therapy to slow down the cognitive deficit by reducing amyloid beta levels in early AD subjects^10^. The acceptance of Aducamab, even if its therapeutic efficacy will remain long debated, has been strongly encouraged by the national agencies, advocacy, groups of patients and family members, showing the power of multiple stakeholders’ actions (Figure 1). The expected cascading effect is the revamp of more public and private investments in R&D, accelerating alternative therapeutic pipelines to stop dementia incidence.

However, to fully achieve this goal, more efforts need to be performed in several directions, including a broader involvement of patients in different activities that pose them at the core in the fight against dementia and ranging from clinical studies design to the realization of awareness campaigns against stigmatization around dementia (Fletcher, 2019; Hand, 2019; Low and Purwaningrum, 2020) to other social events^11^. Fostering meetings between specialists, patients, and their families would enable a trustful network where the latter feel reassured and assisted by the healthcare system and, consequently, more encouraged to proactively cooperate in the advancement of scientific studies, possibly shaping their future with better perspectives. In addition, a large consensus among people would also positively impact another aspect often ignored, namely the necessity of working with heterogeneous patient communities, since worldwide ethnicities differ for genetic traits, culture, and lifestyle (National Research Council (US) Panel on Race, Ethnicity and Health in Later Life, 2004; Chin et al., 2011; Low et al., 2019). All these variables play a crucial role when defining a clinical trial, therefore they must be taken into account to conduct cutting-edge research.

Another advantage when planning clinical studies is represented by the enrichment of the actual preventive strategies, including imaging techniques, fluid biomarkers, classical cognitive tests, and sensory modalities evaluation, with new technologies and instruments to monitor the health status and potentially identify, with greater precision, those subjects at higher risk of developing cognitive impairment. The introduction of digital tests, wearable devices, and VR systems offer the unprecedented opportunity to constantly track physiological parameters and cerebral functions, which can supply a substantial amount of data potentially underlying subtle anomalies occurring in the transitional phases of the pathology. Ultimately, allowing a faster and more accurate personal follow-up (Westerberg et al., 2010; Chester and Rudolph, 2011; Zygouris et al., 2017; Berg et al., 2018; Lucke et al., 2019; Mandryk and Birk, 2019; Piau et al., 2019; Zhavoronkov et al., 2019; Bloniecki et al., 2021; Gielis et al., 2021; Majnarić et al., 2021).

Also in this case, people with dementia can be actively engaged in the implementation and customization of digital technologies by providing inputs and suggestions based on their daily needs.

This interaction triggers a bidirectional benefit, since researchers can successfully accomplish their studies thanks to the valuable feedback of the final users, and patients, with their own experience, can contribute to ameliorate their wellbeing and daily life tasks and also enhance the research quality.

An example is provided by the RADAR-AD project, financed by the Innovative Medicines Initiative (IMI)^12^, where AD patients participate in the planning and realization of innovative user-friendly devices aimed at improving their quality of life. In particular, RADAR-AD takes advantage of mobile and smart home appliances to constantly check individuals’ health condition and adapt supportive measures based on the collected data. This approach has several positive implications since it offers to the subject the possibility to independently monitor the disease continuum and receive faster support by healthcare specialists that can simultaneously analyze multiple parameters and promptly intervene if needed. Moreover, the use of technological interfaces would help the researchers to progress in the understanding of AD in its different phases, from preclinical, to MCI, and late stage, therefore implementing existing platforms, pioneering state-of-the-art systems, and discovering more reliable preventive and therapeutic solutions.

To be successful, this digital evolution needs to be paralleled by the renovation of the healthcare system, pointing at increasing the number of professionals in different categories and enabling their regular training, since only the acquisition of new skills and information would allow a more precise and beneficial effect in managing dementia at all levels, from bench to bedside (Gove et al., 2016; Steiner et al., 2020). In addition, a more impactful participation of policymakers and service providers is of vital importance in this transformation phase (Steiner et al., 2020). The infrastructures’ modernization would also strongly contribute to a more effective patient-centered management satisfying personalized requirements (Galvin et al., 2021).

Overall, these observations emphasize the importance of a joint effort to better understand dementia in its different facets and finally pave the path towards a new era for health monitoring, leading to accurate preventive and therapeutic strategies.

## Conclusion

Dementia is a devastating condition that requires a massive intervention at the international level. For this reason, many organizations and initiatives underline the imminent need for an interdisciplinary coordinated action, where all categories involved in tackling dementia, from researchers and clinicians to patients and caregivers, interact in order to constantly optimize the planning of research projects and clinical studies, as well as to ameliorate the quality of life of those living with dementia. Therefore, to succeed in this challenging task, many aspects need to be considered and better supported, like raising awareness about brain health and aging, to eliminate any discrimination against patients and their families. In addition, challenging inequity and intersectionality in concomitance with efficient informative campaigns on dementia and the technological breakthrough offered by innovation would strongly support the fight against this global health crisis. These concerted societal efforts will fuel the development of groundbreaking technologies, leading towards a new era of personalized health monitoring, where patients can regularly track their psychophysical status with user-friendly devices and will be actively engaged in the advancement of effective and timely therapeutics.

## Author Contributions

EB wrote and reviewed the manuscript. AT, VB-R, and FD’A contributed to the writing and reviewed the manuscript. LA revised the manuscript and prepared Figure 1. All authors contributed to the article and approved the submitted version.

## Conflict of Interest

VB-R is an employee of Virtuleap. The remaining authors declare that the research was conducted in the absence of any commercial or financial relationships that could be construed as a potential conflict of interest.

## Publisher’s Note

All claims expressed in this article are solely those of the authors and do not necessarily represent those of their affiliated organizations, or those of the publisher, the editors and the reviewers. Any product that may be evaluated in this article, or claim that may be made by its manufacturer, is not guaranteed or endorsed by the publisher.

## Footnotes

^1^https://www.who.int/news-room/fact-sheets/detail/dementia; ADI—Dementia facts and figures, 2021

^2^ https://www.fda.gov/drugs/news-events-human-drugs/fdas-decision-approve-new-treatment-alzheimers-disease

^3^ https://www.alzheimer-schweiz.ch/de/startseite

^4^ https://www.alzint.org/resource/world-alzheimer-report-2019/

^5^ https://www.alz.org/help-support/i-have-alz/overcoming-stigma

^6^ https://bebrainfit4life.com/

^7^ https://www.brainhealth.scot/

^8^ https://apps.who.int/iris/bitstream/handle/10665/259615/9789241513487-eng.tif?sequence=1

^9^ https://www.alzint.org/resource/world-alzheimer-report-2019/

^10^ https://www.fda.gov/drugs/news-events-human-drugs/fdas-decision-approve-new-treatment-alzheimers-disease

^11^ https://www.imi.europa.eu/get-involved/patients

^12^ https://www.imi.europa.eu/projects-results/project-factsheets/radar-ad
